# Changing ALK-TKI mechanisms of resistance in re-biopsies of ALK-rearranged NSCLC: ALK mutations followed by SCLC-like histologic transformation: A case report

**DOI:** 10.1016/j.heliyon.2024.e32030

**Published:** 2024-05-31

**Authors:** Qiong He, Xinmin Yu, Junrong Yan, Xun Shi, Hongming Pan

**Affiliations:** a Department of Thoracic Oncology, Zhejiang Cancer Hospital, Hangzhou Institute of Medicine (HIM), Chinese Academy of Sciences, Hangzhou, Zhejiang, China; b Medical Department, Nanjing Geneseeq Technology Inc., Nanjing, Jiangsu, China; c Department of Medical Oncology, Sir Run Run Shaw Hospital, College of Medicine, Zhejiang University, Hangzhou, Zhejiang, China

**Keywords:** NSCLC, Histologic transformation, ALK-TKI, Resistance mechanism, Case report

## Abstract

Anaplastic lymphoma kinase (ALK) inhibitors are the recommended treatment of *ALK*-rearranged non-small cell lung cancer but are prone to eventual drug resistance.

Herein we report a 45-year-old Asian woman diagnosed with *EML4-ALK* rearranged lung adenocarcinoma. Small cell lung cancer-like phenotypic transformation occurred when resistance to crizotinib treatment. Next-generation sequencing was performed and detected an *ALK* rearrangement co-existent with a *TP53* gene mutation in the small cell specimens. The patient had a good response to alectinib with a progression-free survival >7 months. After disease progression, newly emerged *ALK p.G1269A* and *p.L1196 M* gene mutations co-existent with *ALK* rearrangement were detected. The patient had a good initial response to ceritinib treatment, which last for >12 months. After ceritinib failure, however, more complicated mutations within the ALK kinase domain (*p.G1269A*, *p.L1196 M*, newly emerged *p.D1203 N,* and *p.L1122V*) were detected. Ultimately, due to terminal rapid progression and resistance to lorlatinib, the overall survival was nearly 3 years.

Our case showed that next-generation ALK-tyrosine kinase inhibitors (TKIs) may be an appropriate choice after transformation to small cell lung cancer and failure to one ALK-TKI. Sequential biopsies and gene mutation monitoring are important to arrange the sequence of different generation ALK-TKIs. Appropriate sequential therapies may yield a prolonged response with a satisfactory quality of life in patients with advanced *ALK*-rearranged non-small cell lung cancer.

## 1 Introduction

Anaplastic lymphoma kinase (ALK) inhibitors are currently the recommended first-line treatment for *ALK*-rearranged metastatic non-small cell lung cancer (NSCLC) according to the National Comprehensive Cancer Network guidelines. How to select appropriate targeted drugs in patients previously treated with ALK inhibitors, however, has not been established. In fact, the National Comprehensive Cancer Network guidelines recommend that next generation ALK inhibitors can be directly used after ALK inhibitor failure regardless of determining the mechanism underlying drug resistance [[1], [2], [3]].

Previous studies have reported two major mechanisms of drug resistance in the treatment of advanced *ALK*-positive NSCLC failure to ALK inhibitors, as follows: ALK-dependent, such as ALK amplification and a secondary mutation of the ALK kinase domain; and ALK-independent, including bypass activation and phenotypic transformation [[3], [4], [5]]. Due to the relative low incidence of *ALK*-positive NSCLC and rare proportion of phenotypic transformation after ALK-tyrosine kinase inhibitor (TKI) resistance, there are no large clinical study reports that detail the clinical coping strategies and prognosis in this group of patients. Indeed, there are only a few clinical cases reported involving phenotypic transformation failure to ALK inhibitors [[4], [5], [6]]. Drug resistance may occur due to pathologic transformation whether the patient is treated with crizotinib, alectinib, or loratinib. The median time at which small cell lung cancer (SCLC)-like phenotypic transformation occurs is approximately 6 months. *ALK* fusion mutations can be detected in most cases in SCLC components. It has been reported that the management modes of drug resistant transformation into SCLC include chemotherapy, chemotherapy combined with targeted drugs, and direct replacement of next-generation ALK-TKIs [2,6].

**Fig. 1:**
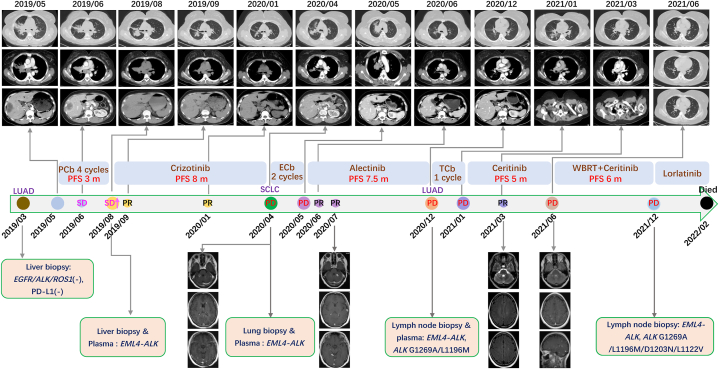
Course of disease with driver mutations, treatment history, and computed tomography images.LUAD, lung adenocarcinoma; SCLC, small cell lung cancer; PCb, pemetrexed and carboplatin; ECb, etoposide and carboplatin; TCb, paclitaxel and carboplatin; SD, stable disease; PR, partial response; PD, progressive disease; PFS, progression-free survival; WBRT, whole brain radiation therapy.

**Fig. 2:**
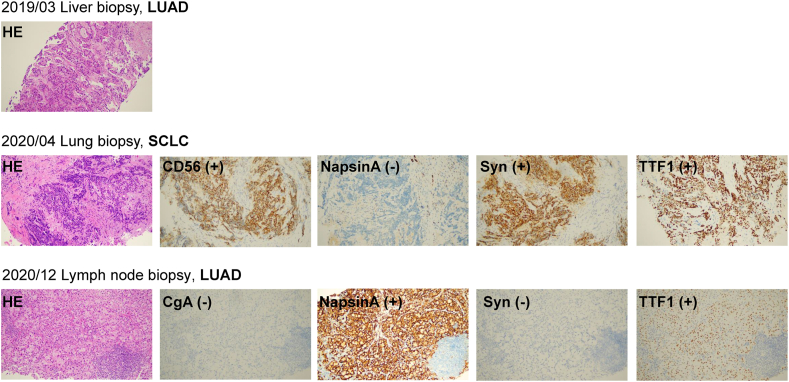
Pathological diagnosis (HE and immunohistochemistry staining) during treatment. HE, hematoxylinandeosin; LUAD, lung adenocarcinoma; SCLC, small cell lung cancer.

Our case involved a patient with *ALK*-positive advanced lung adenocarcinoma that underwent a small cell lung cancer transformation after treatment with a first-generation ALK-TKI (crizotinib) and response to second-generation ALK-TKI (alectinib), followed by drug resistance caused by a secondary mutation in the ALK kinase domain. According to the literature, the corresponding targeted drug (ceritinib) was subsequently used to treat the patient. Good efficacy was achieved, which extended the overall survival. (See Fig. 1, Fig. 2).

### 1.1 Case presentation

A 45-year-old Asian female with clinical condition performance status of 1 when admitted to our hospital for evaluation of a “recurrent cough for >6 months and diagnosed with lung adenocarcinoma for >1 month.” She was admitted to a local hospital for evaluation of a cough in March 2019. Chest and abdominal computed tomography (CT) scans were performed, which revealed right peripheral lobe lung cancer with intrapulmonary, right hilum, mediastinal lymph node, and liver metastases. A cranial CT and emission CT showed no obvious abnormalities. A Pulmonary fiberbronchoscopy examination revealed stenosis of the lumen in the anterior segment of the right superior lobe. The pathologic evaluation of a brush bronchoscopy specimen revealed a small tumor (NSCLC) in the posterior upper lobe of the right lung. An ultrasound-guided biopsy of the liver led to the diagnosis of an adenocarcinoma harboring no gene mutations based on *EGFR*/*ROS*/*ALK* gene direct sequencing.

The patient was referred to the clinic of Zhejiang Cancer Hospita (Hangzhou City, China) for therapy. After completing a staging examination, she was diagnosed with a right lung adenocarcinoma with multiple metastases to the liver, lungs, hilum, and mediastinal lymph nodes (cT3N3M1c IVB [American Joint Committee on Cancer, version 8], *EGFR*/*ROS*/*ALK*/PD-L1-negative).

The patient received four cycles of first-line pemetrexed combined with carboplatin chemotherapy from 17 May to July 18, 2019. Chest and abdominal CT scans showed the best response was stable disease after two cycles (June 26, 2019). The cough had worsened at the follow-up evaluation on August 7, 2019. A CT scan indicated significant tumor progression. The patient declined continued chemotherapy. She was advised to undergo an ultrasound-guided biopsy of the liver again in our clinic. She was diagnosed with an adenocarcinoma harboring an *ALK-EML4* fusion mutation by next-generation sequencing (NGS). The patient was treated with crizotinib (0.25 g twice daily from August 8, 2019 and the cough improved significantly. The efficacy of crizotinib was assessed based on a partial response (PR) after approximately 1 month (September 5, 2019) and the progression-free survival (PFS), which was approximately 5 months.

The patient developed a severe cough in March 2020. Re-examination with an enhanced CT scan of the chest and abdomen showed lesions in the middle and upper lobes of the right lung with distal obstruction that had increased compared to the previous CT scan. The right middle lung bronchus was cut off, suggesting that the lesions had progressed. A fiber-bronchoscopy was suggested and a bronchoscopy biopsy was obtained at a local hospital on March 30, 2020. The pathologic evaluation revealed a neuroendocrine tumor (small cell lung cancer-like). She presented to our clinic for new symptoms (headache and dizziness) in April 2020. Brain magnetic resonance imaging on April 15, 2020 showed multiple abnormal enhanced foci that were consistent with metastatic tumors. Chest and abdominal CT scans on April 15, 2020 indicated stable disease, as before. The brain metastases indicated disease progression. A review of the bronchoscopy biopsy specimen obtained at the local hospital showed that the SCLC in the right upper lobe posterior segment. In addition, the immunohistochemical test results were as follows: TTF-1 (+); NapsinA (−); Sy(+); and CD56 (+). Two cycles of etoposide and carboplatin chemotherapy were administered. As the cough worsened at the beginning of the second ECb cycle, the patient underwent a re-examination earlier than scheduled. Chest and abdominal CT scans on May 9, 2020 showed that the lesions at the bronchial opening of the middle and upper lobes of the right lung were more extensive than before. The lymph nodes of bilateral hilum, left supraclavicular and mediastinal were all enlarged, all of which indicated that the disease had progressed.

Based on previous reports, *ALK* rearrangements can be retained in transformed tumors independent of histologic type [6]. Indeed, *ALK* rearrangements may still exist in the current patient's small cell lung cancer tissues. Gene testing of the bronchoscopy tissues (small cell lung cancer) and blood indicated *ALK-EML4* fusion (plasma, 0.6 %; tissue, 19.5 %) and *TP53* mutations. Based on previous case reports, the patient was eligible for next generation ALK-TKI targeted drug therapy. Oral alectinib (600 mg twice daily) was begun on May 9, 2020. The cough was relieved quickly, and a PR was achieved intra- and extra-cranially.

The patient was periodically evaluated and the results of chest and abdominal CT scans on December 21, 2020 indicated that the right lung lesions were essentially unchanged. Enlarged lymph nodes were noted in the in hilum bilaterally, the left supraclavicular space, and the mediastinum, which were significantly larger than before. The disease progressed again, and the third-line treatment with alectinib had a PFS of approximately 7.5 months. The patient had disease progression but a bronchoscopy showed that the primary lung lesions were stable and no abnormal findings were found. Puncture pathology of a left supraclavicular lymph node indicated a poorly differentiated adenocarcinoma. Immunohistochemical results showed the following: TTF- 1 (+); NapsinA (+); Sy (−); CgA (−). Four-line paclitaxel combined with carboplatin chemotherapy was started on December 28, 2020. However, the cough worsened and severe peripheral neurotoxicity developed. The patient declined further chemotherapy. NGS gene detection was performed on the left supraclavicular lymph node and blood. The results indicated that there was a persistent *EML-ALK* fusion and there was an emergence of new mutations (*ALK p.G1269A*, *L1196 M*, and *TP53*). According to previous reports, the drug resistance might be caused by an *ALK* downstream kinase domain secondary mutation. It has been reported that *ALK p.G1269A* and *p.L1196 M* mutations had different sensitivities to different ALK inhibitors. ceritinib might be a sensitive targeted drug [2,3]. Therefore, she began to receive fifth-line ceritinib (450 mg daily) oral targeted therapy beginning on January 19, 2021. After one week of ceritinib, the cough improved significantly. On the 50th day (March 18, 2021), the patient underwent chest and abdominal CT examinations, indicating that enlarged lymph nodes in the hilum bilaterally, left supraclavicular space, and mediastinum were significantly reduced. In June 2021, The patient returned with complaints of paroxysmal dizziness and discomfort without vomiting or headaches. A review of craniocerebral magnetic resonance imaging indicated tumor progression. The PFS on ceritinib was 5 months. Chest and abdominal CT scans indicated that the extracranial lesions were stable. The patient received whole brain radiotherapy (WBRT, 30 Gy in 10 fractions) beginning on June 19, 2021. After radiotherapy, the dizziness resolved. Oral ceritinib was continued until November 2021, at which time the dry cough worsened. The patient's clinical condition rapidly switched to a performance status of 2 when coming back in January 2022. Chest and abdominal CT scans indicated slow tumor progression. A pathologic evaluation of a left supraclavicular lymph node re-puncture confirmed adenocarcinoma. NGS gene test results showed an *EML-ALK* fusion, and *ALK p. G1269A, p.L1196 M*, and *TP53* mutations, as well as newly emerged *ALK p.D1203 N* and *ALK p. L1122V* mutations. In January 2022, she began to take loratinib but her cough worsened with hemoptysis. She died in early February 2022 with tumor progression and interstitial pneumonia.

## 2 Discussion

ALK is a potent oncogenic driver in the development and progression of, and ALK-TKIs provide survival benefits for patients with ALK-positive advanced NSCLC. However, almost all patients will eventually developed resistance. There are two main molecular modes of resistance associated with ALK inhibitors: ALK-dependent and ALK-independent. ALK-dependent resistance includes resistance mutations in the ALK kinase domain and an increase in *ALK* copy number. For example, *ALK p. G1202R, L1196 M* and *G1269A* mutations are common resistant mutations. The earliest identified ALK resistance mutation was gatekeeping mutation, such as *ALK p. L1196 M*, which can lead to first-generation TKI, crizotinib resistance. Another class of mutations, such as *ALK p. G1202R*, accounting for about 50 % of second-generation ALK-TKIs resistance [7].The ALK inhibitor resistant mutations are well-studied and the corresponding ALK-TKIs are sensitive to specific kinase domain mutations. AlK-independent resistance includes bypass activation, such as *MET* and *RAF* pathway activation or phenotypic transformation, which can be transformed into SCLC and squamous cell carcinoma. In these transformed patients, SCLC type transformation is the most common type of drug resistance [[2], [3], [4], [5], [6]]. Although primary SCLC is sensitive to ECb chemotherapy, the transformed SCLC type is less sensitive after TKI resistance [[7], [8]]. Re-examination of NGS results indicated that the small cell component still had an *ALK* rearrangement. Previous reports with a few references suggested that an *ALK* fusion was still present in SCLC components, indicating that tumor might be response for the next generation of ALK inhibitors. Indeed, the patient presented herein received significant clinical benefit from the second-generation ALK inhibitor, alectinib, especially for the brain lesions. The subsequent resistance to second-line alectinib was demonstrated by NGS, which indicated another resistance mechanism, a new secondary mutation in the ALK kinase domain. A treatment duration of nearly 1 year was obtained by switching to ceritinib based on the previous drug sensitivity results. Additional mutations in the ALK kinase domain were developed with re-resistance to ceritinib. Our patient had a very rare *ALK*-rearrangement lung adenocarcinoma and received ALK inhibitors and developed SCLC transformation and secondary mutations in the ALK kinase domain with drug resistance. The precise application of each ALK-TKI targeted therapy resulted in survival benefit to this patient.

This patient was diagnosed with a right NSCLC with multiple metastases in the liver, lungs, hilum, and mediastinal lymph nodes (staging cT3N3M1cIVB) with *ALK*-positive and PD-L1-negative expression. After receiving chemotherapy and multiple ALK inhibitors treatment, drug resistance caused by SCLC transformation and secondary mutation of the ALK kinase domain occurred. Subsequent adjustment of targeted ALK therapy based on relevant clinical evidence resulted in survival benefit for this patient [7,9,10]. This case provides evidence for the subsequent research on the mechanism of drug resistance and coping strategies in *ALK*-positive NSCLC-targeted therapy. However, there are still several points worth considering. One is that the initial gene test of this patient suggested a co-mutation of *TP5*3/ALK. There is no evidence that first-line treatment of NSCLC with *ALK* fusion and other coexisting gene mutations requires a stronger treatment regimen. Indeed, it has been reported that an ALK inhibitor combined with anti-vascular targeted therapy is effective [11,12]. Furthermore, how to select treatment for SCLC phenotype transformation failure to targeted therapy for *AlK*-fusion NSCLC needs to be determined. Whether the gene status of the SCLC component can be directly detected and whether the gene status can be directly converted into the next generation of targeted drugs remains to be studied. Currently, guidelines recommend directly replacing the next generation TKIs after resistance to one ALK-TKI. Due to the bottleneck of detection technology, few cases can detect the precise mechanism leading to drug resistance mechanism. Multiple re-biopsies and large panel NGS detection can identify the pathologic type and genes status to choose accurate treatment to improve the quality of life and prolong survival.

## Ethics statement

The authors confirm that they have obtained written consent from the patient to publish the anonymized case details and images.

## Data availability statement

Data associated with this study has not been deposited into a publicly available repository. Data will be available upon request.

## Funding

None declared.

## CRediT authorship contribution statement

**Qiong He:** Writing – original draft, Conceptualization. **Xinmin Yu:** Validation, Investigation, Conceptualization. **Junrong Yan:** Software, Visualization. **Xun Shi:** Data curation, Validation. **Hongming Pan:** Writing – review & editing, Supervision, Conceptualization.

## Declaration of competing interest

The authors declare that they have no known competing financial interests or personal relationships that could have appeared to influence the work reported in this paper.
